# Oxidative stress in gut T_H_17 cells makes mice susceptible to bacterial infection

**DOI:** 10.1097/IN9.0000000000000049

**Published:** 2024-11-13

**Authors:** Simon O’Shaughnessy, David K. Finlay

**Affiliations:** 1 School of Biochemistry and Immunology, Trinity Biomedical Sciences, Trinity College Dublin, Ireland; 2 School of Pharmacy and Pharmaceutical Sciences, Trinity Biomedical Sciences, Trinity College Dublin, Ireland

**Keywords:** T_H_17, CD4, IL22, IL17, mitochondria, reactive oxygen species, mTORc1, gastrointestinal infection with *Citrobacter rodentium*, glutathione, glutamate-cysteine ligase catalytic subunit, mitochondrial transcription factor A

## Abstract

A recent paper published in *Cell Metabolism* in August 2024 by Dirk Brenner’s laboratory highlights the importance of effectively managing reactive oxygen species (ROS) in gut T_H_17 T cells for minimizing the damage caused by intestinal bacterial infection. This commentary will discuss the control of cellular ROS by glutathione and the emerging understanding that neutralizing ROS in immune cells is essential for the individualized functions of different immune subsets. In the case of this study, managing ROS within T_H_17 cells in the gut was shown to be essential to sustain the production of IL22 cytokine to maintain gut homeostasis in response to bacterial infection.

A side product of oxygen-dependent cellular respiration is the production of ROS in the mitochondria. Mitochondrial respiration occurs in the inner mitochondrial membrane and is mediated by the electron transport chain (ETC) comprising four complexes (I–IV) and adenosine triphosphate (ATP) synthase. Under normal conditions, electron transfer in the ETC leads to the generation of ATP and the reduction of oxygen to water. However, during inflammation or cellular stress, electrons can leak from complex I and complex III leading to the reduction of molecular oxygen to superoxide (O_2_^−^) serving as a precursor for other ROS including hydrogen peroxide (H_2_O_2_) by the enzyme superoxide dismutase (SOD). These highly reactive molecules play crucial roles in cells by participating in redox signaling including supporting T cell receptor signaling and regulation of gene expression ^[1]^. However, excess ROS have the potential to cause widespread damage within the cell, including disrupting the architecture and function of mitochondria and damaging genomic DNA in the nucleus. As a result, cells have evolved anti-oxidant mechanisms to neutralize excess ROS that include the GSH anti-oxidant system (Figure 1A). The GSH system functions by producing reduced GSH to neutralize ROS, primarily H_2_O_2_ into H_2_O. There is evidence that increased ROS and mitochondrial damage are associated with T cell dysfunction in various diseases, including chronic viral and bacterial infection and cancer ^[2–5]^. For this reason, there is an interest in understanding the relative importance of GSH, and its control of ROS production, for the metabolism and function of different lymphocyte subsets. One way to study this has been through the use of transgenic mice where one of the key enzymes for the synthesis of GSH, such as glutamate-cysteine ligase catalytic subunit (Gclc), is deleted in cells of interest using site-specific recombination technology (Cre-LoxP recombination). Gclc encodes for a subunit of the enzyme glutamate-cysteine ligase, which catalyzes the conjugation of glutamate and cysteine to form γ-glutamylcysteine acting as the first and rate-limiting step of GSH synthesis. In this study by Bonetti et al, they use Cre recombinase under the control of the CD4 promoter to delete Gclc in T cells (Gclc^flox^CD4^Cre^) ^[6]^.

Bonetti et al explored the importance of GSH and oxidative stress in T cells of the gut and how this contributes to the immune response during bacterial gastrointestinal infections. Previous studies using these mice showed that deletion of Gclc did not affect the development of T cells within the thymus nor initial activation in response to anti-CD3 + anti-CD28 stimulation in vitro. However, ineffective management of ROS levels compromised T cell metabolic reprogramming conferring resistance to experimental autoimmune encephalomyelitis, a CD4^+^ T cell-driven murine model of multiple sclerosis ^[7]^. Bonetti et al now show that Gclc^flox^CD4^Cre^ are markedly susceptible to gastrointestinal (GI) infection with *Citrobacter rodentium;* Gclc^flox^CD4^Cre^ mice had extensive intestinal damage and most mice succumbed to infection by 20 days post infection. These data showed that GSH production in T cells is essential for protection against GI infections. Analysis of the colonic lamina propria cells revealed that the predominant T cell subset responding to *C. rodentium* infection in colonic lamina propria cells of the gut was a CD4^+^ T_H_17 subset producing both IL17 and IL22 cytokines. Strikingly, in Gclc^flox^CD4^cre^ mice, this population of cells was absent when infected with *C. rodentium* implicating GSH as a regulator of IL17 and IL22 in CD4^+^ T_H_17 cells. As mice lacking IL22 are unable to clear *C. rodentium* and succumb to the infection ^[8]^, the authors considered if a lack of IL22 production by this subset of T_H_17 cells underpinned the phenotype of *C. rodentium* Gclc^flox^CD4^Cre^ mice. First, they treated infected mice with recombinant IL22 and this intervention prevented intestinal damage, weight loss, and death. This IL22 requirement was attributed to the regulation of intestinal permeability; there was a loss of epithelial tight junctions in infected Gclc^flox^CD4^Cre^ mice that were rescued by the administration of recombinant IL22. Notably, in addition to CD4^+^ T_H_17 cells, type 3 innate lymphocytes (ILC3) also produce IL22 in the gut ^[9]^. The lymphoid tissue inducer (LTi) subset of ILC3s expresses CD4 and is thus targeted by CD4Cre-driven Gclc deletion. To demonstrate that the observed IL22-dependent phenotype was due to CD4^+^ T_H_17 cells rather than ILC3s, the authors analyzed caecal LP CD4^+^ LTi cells from Gclc^flox^CD4^Cre^ and Gclc^flox^ mice at days 4 and 7 post infection revealing no defect in LTi cell frequency and cytokine production in contrast to IL17^+^IL22^+^ T_H_17 cells. To ascertain if IL22 production in T cells was the key factor underpinning the observed phenotype in Gclc^flox^CD4^Cre^ mice, the researchers performed a rescue experiment. Transgenic mice were generated that constitutively expressed IL22 or IL17A only in T cells of Gclc^flox^CD4^Cre^ mice. Transgenic expression of IL22 but not IL17A was sufficient to protect Gclc^flox^CD4^Cre^ mice from *C. rodentium* infection demonstrating IL22 as the primary mediator of protection. As shown here by Bonetti et al ^[6]^, the response to *C. rodentium* infection is primarily driven by T_H_17 immunity. It will be important to investigate the translation of these findings to other experimental models of IBD that are mediated by other T helper subsets.

Mechanistically, in vitro cultured T_H_17 cells from Gclc^flox^CD4^Cre^ mice had clear evidence of increased mitochondrial ROS and impaired mitochondrial function, including reduced mitochondrial respiration and mitochondrial membrane polarization (Figure 1B). Indeed, deletion of Gclc in other types of cells causes significant mitochondrial dysfunction, including hepatocytes, neurons, and follicular B cells ^[10–12]^ but this is not the case for all T cell subsets. Deletion of Gclc in Tregs using a FoxP3-Cre resulted in an overall increase in mitochondrial respiration in regulatory T cells highlighting the subset-specific response to oxidative stress ^[13]^. In contrast to T_H_17 cells, Gclc depletion in Tregs promotes mammalian target of rapamycin complex 1 (mTORc1) signaling by enhancing serine and 1-carbon metabolism flux. This disparity in response may relate to different metabolic requirements that support subset-specific functions, elevated mitochondrial activities that certainly influence the propensity for ROS production, and the activity of other anti-oxidant enzymes including catalase, SOD, peroxiredoxins, and thioredoxins ^[14]^. Indeed, cytosolic thioredoxin-1 is important in rapidly proliferating T cells to support redox reactions essential for nucleotide synthesis ^[15]^. Further, T_H_2 and T_H_17 differentiation is restricted in SOD3 knockout CD4 T cells ^[16]^. Bonetti et al show that the observed mitochondrial dysfunction of in vitro differentiated Gclc^flox^CD4^Cre^ T_H_17 cells was associated with reduced expression of mitochondrial encoded genes for the ETC. This defect in mitochondrial ETC genes was associated with loss of mitochondrial transcription factor A (TFAM) expression in Gclc mutant mice suggesting a regulatory feedback loop between GSH-dependent ROS scavenging and mitochondrial fitness. Considering the regulatory role of TFAM in mitochondrial function and ROS as a byproduct of this process, the interrelationship between ROS production and TFAM expression offers a potential metabolic checkpoint. The reduction of TFAM expression during oxidative stress has been reported in HK2 cells in a model of ischemic acute kidney injury ^[17]^. Conversely, in a study using rat hepatoma cells, mitochondrial oxidative stress promoted TFAM expression by promoting the Akt-mediated phosphorylation and nuclear translocation of nuclear respiratory factor 1 ^[18]^. Considering the diverse responses of T_H_ subsets to ROS further work is required to determine whether the observed regulatory feedback loop is specific to T_H_17 cells. The expression of these genes and the overall mitochondrial fitness of Gclc^flox^CD4^Cre^ T_H_17 cells were rescued when cells were cultured with the anti-oxidant *N*-acetylcysteine (NAC). Indeed, administration of NAC in the drinking water significantly reduced the mortality and GI phenotype of *C. rodentium*-infected Gclc^flox^CD4^Cre^ mice. Mitochondrial dysfunction observed for in vitro differentiated Gclc^flox^CD4^Cre^ T_H_17 cells was associated with alterations in cellular signaling pathways, including the inhibition of phosphoinositide-3-kinase and mTORc1 signaling. However, the cause of mitochondrial dysfunction requires further investigation; specifically whether enhanced ROS directly influences mitochondrial fitness in addition to reduced TFAM and mtDNA gene expression. Furthermore, the mechanism by which ROS influences TFAM expression requires further clarification. Interestingly, while there was a reduction in IL22 protein level in T_H_17 cells, the level of Il22 mRNA was not affected. Overall, the authors propose a mechanism where loss of redox control in Gclc^flox^CD4^Cre^ T_H_17 T cells leads to mitochondrial dysfunction and inhibition of specific signaling pathways resulting in reduced IL22 protein translation and release. Post-transcriptional regulation of cytokine production at the level of translation has been reported in different contexts. For example, double knockout of the mTORc1 targets EIF4EBP1 and EIF4EBP2 has been shown to control interferon production by plasmacytoid dendritic cells through EIF4e regulation of IRF7 expression ^[19]^. In T cells, the glycolytic enzyme GAPDH has been shown to bind to the 3’UTR of Il2 and Ifng mRNA and repress the translation of these transcripts ^[20]^. Proinflammatory tumor necrosis factor (TNF) is regulated at multiple levels including the control of TNF protein translation ^[21]^.

This detailed study by Bonetti et al has furthered our understanding of the importance of redox balance in specific T cell subsets and the consequences when this balance is disturbed. In the case of *C. rodentium* infection, increased oxidative stress due to loss of GSH in CD4^+^ T_H_17 cells leads to impaired intestinal barrier function during GI bacterial infection on account of defective T cell production of IL22. It will be interesting to consider if this mechanism affects human susceptibility to GI infections and how this axis might be targeted therapeutically. CAR T cell approaches offer an exciting therapeutic modality to eliminate autoreactive lymphocytes or increase the immunosuppression of Treg cells to treat autoimmunity and gut inflammation ^[22,23]^. This study demonstrates how a deficiency in IL22 disrupts the integrity of the intestinal epithelium. Therefore, modulation of CAR T cell therapies to allow the cells to produce IL22 might enhance the efficacy of these therapies to mediate the repair of the diseased gut. Further, randomized controlled clinical trials have demonstrated a positive effect of administering NAC in reducing disease activity and the maintenance of remission in patients with ulcerative colitis ^[24,25]^. It would be interesting to explore further if this effect involves improved metabolic and functional fitness of gut resident T_H_17 cells.

## Figures and Tables

**Figure 1:**
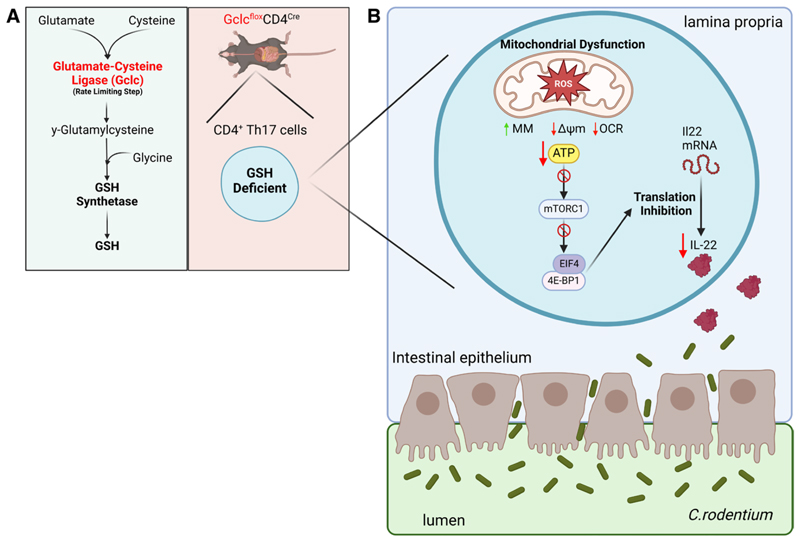
Gclc loss in gut CD4 T_H_17 cells impairs IL22-mediated gut homeostasis. (A) Glutamate-cysteine ligase (Gclc) is the rate-limiting step in the de novo synthesis of glutathione (GSH) from the amino acids glutamate, cysteine, and then glycine (left). Specific deletion of Gclc in all T cells can be achieved by crossing mice where the *Gclc* gene is flanked by loxP sites with mice expressing Cre recombinase under the control of the CD4 promoter to generate Gclc^flox^CD4^Cre^ mice. All T cells, including CD4 T_H_17 cells, will be GSH deficient in these mice (right). (B) Gclc^flox^CD4^Cre^ mice are susceptible to *C. rodentium* infection in the gut. In these mice, gut CD4 T_H_17 cells exhibit increased reactive oxygen species (ROS) and mitochondrial dysfunction; T_H_17 cells have increased mitochondrial mass (MM), decreased mitochondrial membrane potential (ΔΨm), and reduced respiration (OCR). Reduced ATP levels and reduced activity of the mTORc1 kinase result in defective translation of Il22 cytokine mRNA, a cytokine essential for regulating the integrity of the intestinal epithelium. The figure was generated using BioRender (https://www.biorender.com/). ATP, adenosine triphosphate; mTORc1, mammalian target of rapamycin complex 1; OCR, oxygen consumption rates.
